# Relationship between engagement and level of functional status in older adults

**DOI:** 10.1177/2050312117727998

**Published:** 2017-09-04

**Authors:** Thomas A Dombrowsky

**Affiliations:** College of Nursing and Health Innovation, The University of Texas at Arlington, Arlington, TX, USA

**Keywords:** Engagement, older adults, activity theory, functional status

## Abstract

Functional status is an important component of quality of life for older adults and for their caregivers. Factors associated with level of functional status include age, comorbidity, cognitive status, depression, social support, and activity. Of the types of activity linked with functional status, the strongest evidence is for physical exercise, with weaker evidence for social and productive activity. Engagement is a construct including motivation, commitment, and participation.

**Objective:**

The objective of this study was to determine whether or not engagement is associated with level of functional status after controlling for age, comorbidity, and depression among older adults.

**Methods:**

In all, 92 older adults were recruited from senior living centers and a university-based senior exercise group. The main independent variable was engagement. Other independent variables were age, comorbidity, and depression. The dependent variable was level of functional status. Two different measures of level of functional status were used: the Katz Activities of Daily Living Index and the Lawton–Brody Instrumental Activities of Daily Living Scale.

**Results:**

Engagement was a significant predictor of Lawton–Brody Instrumental Activities of Daily Living Scale level of function in the Lawton–Brody model (odds ratio: 1.147 (1.011–1.302)) and age was a significant negative predictor (odds ratio: 0.838 (0.769–0.914)). There were no significant predictors in the Katz model.

**Conclusion:**

Engagement is a significant predictor of perfect Lawton–Brody Instrumental Activities of Daily Living Scale score after controlling for age, comorbidity, and depression. Health-care professionals should consider including engagement in their assessment of older clients, whether using formal instruments such as the Engagement with Meaningful Activity Survey or assessing informally.

## Introduction

Wang defines functional status as the level of ability to do “activities performed by an individual to realize the needs of daily living in many aspects of life including physical, psychological, spiritual, intellectual, and roles.”^1^ It is typically measured by assessing ability to do activities of daily living (ADLs) and instrumental activities of daily living (IADLs) independently.^2,3^ Engagement is a construct consisting of three components: motivation, commitment, and participation in a given activity.^4^ In this article, literature concerning the relationship between activity and the functional status of older adults is briefly reviewed and a study of the relationship between engagement and level of functional status is reported.

### Functional status of older adults

The portion of the US population over 60 years of age is expected to be 27% of 400 million by the year 2050.^5^ Currently, only 2.3% of the US adult population requires assistance with ADLs, but this number rises to 3.4% among adults aged 65–74 years and 12% for those over 75 years.^6^ In 2014, there were an estimated 46.2 million older adults in the United States,^7^ about 680,000 of whom are in residential care communities and 1.2 million in nursing homes.^8^

Functional status is one of the most important determinants of quality of life for older adults.^9–11^ Functional status is usually operationalized as the degree of dependence a person experiences in performing ADLs and IADLs.^2,3^ The ADLs typically included in functional status assessment are eating, transfers, continence, toileting, bathing, and dressing.^12^ More sensitive indicators of functional status include mobility and IADLs such as shopping, meal preparation, telephone use, housekeeping, laundry, arranging transportation, and managing medications and finances.^2^

In addition to being related to quality of life, functional status also has an important relationship to physical health and longevity.^13^ Functional decline is defined as a need for assistance in at least one ADL.^13^ Functional decline is thus a level of functional status, as is intact functional status. Decourcelle et al. did a hospital-based study of 272 Canadians with an acute coronary event and found that those with functional decline were more likely to die than those without functional decline. They found hazard ratios for mortality of 3.63 at 6 months and 2.69 at greater than 6 months from the coronary event.

Functional decline is also a financial burden. Current annual long-term care costs are estimated at 147.4 billion dollars for the United States.^14^ A study of US Alzheimer’s patients reported an estimated average increase of US$1406 annually in direct medical care costs and US$3333 in total costs per patient for each one point increase in the Blessed Dementia Rating Scale.^15^ Investigators in Ireland estimated a 6-month increase of 796 euros for each one point decline in the Katz ADL Scale.^16^

Functional decline affects family members, who frequently become caregivers as a result. In a primary practice-based study of 165 informal caregivers, Nichols et al.^17^ found that new caregivers tended to request information about the technical aspects of caregiving, but as time went on caregivers tended to focus more on the emotional aspects. As a group, the caregivers had high rates of depression and they tended to neglect their own health. Findings of a matched control study of 37 caregivers were that the caregivers had greater rates of depression and anxiety and lower sense of belonging, positive affect, and sleep efficiency than their controls.^18^

### Engagement

Lequerica and Kortte^4^ define engagement as the level of motivation, commitment, and participation that a person has toward an activity. The important things to measure in assessments of engagement are the individuals expressed attitude toward the activity as well as acknowledgment of need and actual participation in the activity.^19^ Some investigators measure engagement with direct observations,^20^ while others use questionnaires.^21–23^

Factors which are known to be related to level of functional status include age,^24^ comorbidity,^25^ cognitive status, depression,^26^ social support,^27^ and activity.^28^ There has been little study of the relationship between engagement and level of functional status. One study of orthopedic rehabilitation patients reported better level of functional status for more engaged patients.^19^ Cohen-Mansfield et al.^29^ found a negative correlation between level of functional status and engagement among nursing home residents with dementia. The purpose of the study reported here is to investigate the relationship between engagement and level of functional status in older adults. The research question is as follows: Controlling for age, comorbidity, and depression, is engagement related to level of functional status among cognitively intact older adults?

### Framework

**Figure 1.**
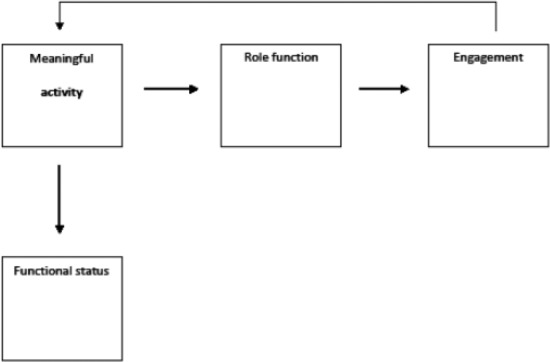
Activity study framework.

The framework for this study is based on activity theory. Activity theory holds that older adults generally desire to maintain their accustomed activities and that doing so is beneficial for them both physically and mentally.^30^ The framework includes the concepts of engagement, role function, meaningful activity, and functional status. According to the framework, roles are activated by meaningful activity. This activation results in engagement, which leads to a self-reinforcing cycle. Improved or maintained functional status is a byproduct of this cycle. The relationships between the concepts of the framework are illustrated in Figure 1.

### Age

One of the factors known to be associated with level of functional status is age.^24^ A multi-year study of the prevalence of functional decline in Sweden reported rates of 10%–12% for participants in the 81–84 year range compared with 32%–40% for those between 90 and 99 years.^31^ Cumulative life-time adversity and depression are moderator variables for age in the trajectory of functional decline.^32^

### Comorbidity

Comorbidities are also associated with functional decline.^25^ Comorbidities of particular interest include arthritis, hypertension, heart disease,^33^ obesity,^34^ liver disease,^35^ stroke, and chronic obstructive lung disease.^36^

### Cognitive function

Impaired cognitive ability correlates with functional decline.^26^ Executive function is especially important for maintaining level of functional status,^37^ as are attention and verbal memory.^38^ In this study, cognitive function was not measured, but it was controlled for by only admitting participants whose cognitive function was sufficient to understand and verbalize the contents of the consent document.

### Depression

The relationship between depression and level of functional status is complex. Some studies suggest that depression leads to functional decline,^32,39,40^ but others suggest that functional decline leads to depression.^41–43^

### Social support

Higher social support is associated with increased level of physical activity^44^ as well as with higher level of functional status.^27^ Social support may also be a moderator between functional status and depression.^45,46^ McLaughlin et al.^27^ found that the participant’s satisfaction with social support is more predictive of level of functional status than the size of the social support network itself.

### Activity

Of the various types of activity, physical exercise is the activity most strongly linked to better level of functional status.^28,47^ Self-efficacy is a mediator between physical exercise and level of functional status.^48^ The benefit of physical exercise for level of functional status is dose related.^49^

Solitary cognitive activity, such as reading or doing crossword puzzles, is associated with a slower trajectory of cognitive decline in older adults with Alzheimer’s disease.^50^ Social activity is associated with higher level of functional status.^51,52^ Soubelet found that social activity is a mediating variable between age and cognitive function.^53^

Research on productive activity, such as paid employment, volunteer work, or informal childcare, is mixed. Hsu^54^ found that although paid work was protective against mortality, for women, unpaid productive activity such as childcare was associated with increased mortality, but unpaid productive activity for men was associated with lower rates of cognitive decline.

### Engagement

Engagement is a construct with three components: the degree of motivation, participation, and commitment which a person has toward an activity.^4^ Meaningful activity is a closely related concept which consists of actions which the individual considers important.^55^ Activity which is congruent with a person’s accustomed roles is more likely to be meaningful.^56^

## Method

A power analysis determined that a sample of 100 would give 83% power for R^2^ = 0.08. This was considered adequate to detect any clinically significant relationship. This value for R^2^ translates to a value of F^2^ = 0.087. Although the intention was to recruit 100 participants, in fact, only 92 older adults were recruited from three comprehensive senior living communities in a large southern metropolitan area and from a university-based senior exercise group. The study was approved by the university institutional review board (IRB). Participants had to be 65 years or older, have intact decisional capacity, and be able to understand the written consent. Decisional capacity was assessed by having the potential participant read the consent or have it read to her/him and then asking the participant to describe her/his understanding of the purpose and activities of the study. Study procedures included written survey forms and oral interviews. No participants used a proxy to respond to the survey items, but two participants did ask the investigator to read the items to them so that they could respond verbally. All study procedures were conducted in one session at the recruitment site. Variables of interest were, age, engagement, comorbidity, depression, and level of functional status. Participants were compensated for their time and trouble with a small gift card.

### Instruments

The instruments used were the Engagement with Meaningful Activity Survey (EMAS), the Functional Comorbidity Index (FCI), the Geriatric Depression Scale–Short Form (GDS-SF), the Katz Index of Independence in ADLs (Katz), and the Lawton–Brody IADL Scale (Lawton–Brody). The independent variable of interest was engagement. Age, comorbidity, and depression were control variables.

The engagement variable was measured by the Engagement in Meaningful Activities Survey (EMAS).^57^ This test had a Cronbach’s alpha score of 0.84 and a test–re-test reliability of 0.69 in the work of the original researchers. Concurrent validity was supported by EMAS scores correlating positively with measures of life satisfaction and purpose in life.^55^ The scores correlated negatively with depression. Possible EMAS scores range from 12 to 48 with higher scores indicating greater engagement.

The FCI is a measure of comorbidities specifically designed with functional status as a primary outcome variable.^58^ It consists of yes/no questions to a list of 18 different diagnoses. The instrument was developed using data from the Canadian Multi-Centre Osteoporosis Study and the National Spine Network database. FCI scores can range from 0 to 18. Higher scores indicate more comorbidities.

The GDS-SF is a 15-question version of the original 30-question Geriatric Depression Scale.^59,60^ Cronbach’s alpha over various studies has ranged from 0.76 to 0.83, and test–retest reliability was an average of 0.85. A comparison of GDS scores with diagnosed depression resulted in a sensitivity rate of 78.7% and specificity of 93.9%.^61^ The range for GDS-SF scores is 0–15 with higher scores indicating greater depression.

The Katz is an assessment of basic functional areas such as dressing, transferring, and continence.^62^ The Katz is predictive of discharge to home, and it correlates well with measurements of home confinement and mobility.^63^ In contrast, the Lawton–Brody focuses on higher cognitive level self-care areas such as telephone use and managing transportation needs.^64^ Reported measures of reliability and validity for the Lawton–Brody include inter-rater reliability of 0.85 and concurrent validity demonstrated by high correlation with other tools for measuring IADLs, emotional status, social adjustment, and health.^65^ Katz scores can range from 0 to 6, and Lawton–Brody scores have a range of 0–8. Higher scores on both of these instruments indicate greater functional independence.

### Analyses

Descriptive statistics were compiled for age, gender, Hispanic identity, race, and marital status. Missing data were imputed using multiple imputation. Two regression models were fit, one with the Katz as the dependent variable and the other using the Lawton–Brody. Statistical packages used were R core software^66^ along with the lawstat^67^ and mice^68^ packages for R.

The research variables were tested for normality using the Shapiro–Wilk test and for homoscedasticity using the Levene test. Age was found to be normally distributed, as were the squared values of the EMAS scores, but the rest of the variables were skewed. The Lawton–Brody was very heteroscedastic. Rethinking the situation, the researchers decided that anything less than a perfect score on the functional status measures indicated some degree of dependence. This fits well with Decourcelle et al.’s^13^ definition of functional decline as being the need for assistance on one or more ADLs. The researchers in this study expanded this definition to include need for assistance in one or more IADLs.

Katz and Lawton–Brody scores are not truly interval/ratio data even though they are often treated as such. One could go down a point in the Katz for incontinence or one could go down a point for needing assistance with eating. These two very different situations could result in the same Katz score. The researchers decided that the broader concepts of dependence and independence were a better way to handle this situation. For these reasons, the decision was made to dichotomize the outcome variables (Katz score and Lawton–Brody score) and to use logistic regression in the models.

The resulting dichotomized variables were either a perfect score or a less-than perfect score for each instrument. Dichotomization can be justified when the underlying structure of the data is itself categorical.^69^ MacCullum et al. give the example of a study where data are collected on numbers of cigarettes smoked per day. There would be a large cluster of data at the zero point with the rest of the data distributed over a range from 1 to 20 or more. The variable in question could be dichotomized as “smokers” and “nonsmokers.” The distinction between being functionally independent and having some degree of dependence is similar to the distinction between being a smoker and being a nonsmoker.

## Results

### Sample description

Participants were recruited from three different senior living centers and from one university-based senior exercise group. A total of 95 participants were recruited, but 3 were excluded for failure to meet the age criterion. Data were incomplete on one or more instruments for eight participants. Sample demographics are given in Table 1. Means for the main variables are given in Table 2.

**Table 1.** Sample demographics.

Characteristic	n	%	Characteristic	n	%
Female	68	74%	Assisted living	2	2%
Male	24	26%	Independent living (senior center)	57	62%
White	88	96%	Community dweller	33	36%
Black	2	2%	Divorced	17	18%
American Indian	1	1%	Married	42	46%
Did not answer race question	1	1%	Single	4	4%
Hispanic	1	1%	Widowed	37	40%
Not Hispanic	69	75%	No answer to marital question	2	2%
No answer to Hispanic question	22	24%			

**Table 2.** Means and ranges for main variables.

Variable	Mean (SD), n	Range
Age (years)	80.18 (8.01), n = 92	65–101
EMAS	39.57 (4.54), n = 88	27–48
FCI	4.07 (2.23), n = 92	0–10
GDS-SF	1.61 (1.76), n = 88	0–10
Katz	5.67 (0.56), n = 91	3–6
Lawton–Brody	7.47 (1.06), n = 92	3–8

SD: standard deviation; EMAS: Engagement with Meaningful Activity Survey; FCI: Functional Comorbidity Index; GDS-SF: Geriatric Depression Scale–Short Form; Katz: Katz Activities of Daily Living Index; Lawton–Brody: Lawton–Brody Instrumental Activities of Daily Living Scale.

### Models

In all, 64 participants scored 1 for the dichotomized Katz, and 65 scored 1 for the dichotomized Lawton–Brody. Two models were fit. Independent variables for both models were age, EMAS score, FCI score, and GDS-SF score. The logistic regression model using the Katz as the outcome variable did not have any significant predictors, but age and EMAS score were significant in the model for the dichotomized Lawton–Brody. Both models are summarized in Tables 3 and 4.

**Table 3.** Logistic regression model with dichotomized Katz score as dependent variable.

	Crude OR	Adjusted OR	p
Age (years)	1.002 (0.947–1.062)	0.985 (0.923–1.053)	0.660
EMAS score	0.926 (0.826–1.030)	0.922 (0.814–1.035)	0.180
FCI score	0.850 (0.685–1.043)	0.837 (0.664–1.040)	0.115
GDS-SF score	1.063 (0.818–1.439)	1.204 (0.883–1.766)	0.286

Katz: Katz Activities of Daily Living Index; OR: odds ratio; EMAS: Engagement with Meaningful Activity Survey; FCI: Functional Comorbidity Index; GDS-SF: Geriatric Depression Scale–Short Form.

**Table 4.** Logistic regression model with dichotomized Lawton–Brody score as dependent variable.

	Crude OR	Adjusted OR	p
Age (years)	0.860 (0.793–0.922)	0.847 (0.767–0.919)	<0.001
EMAS score	1.087 (0.980–1.212)	1.183 (1.037–1.376)	0.018
FCI score	0.927 (0.755–1.134)	0.889 (0.681–1.147)	0.370
GDS score	0.902 (0.696–1.172)	1.090 (0.794–1.521)	0.587

Lawton–Brody: Lawton–Brody Instrumental Activities of Daily Living Scale; OR: odds ratio; EMAS: Engagement with Meaningful Activity Survey; FCI: Functional Comorbidity Index; GDS-SF: Geriatric Depression Scale–Short Form.

Because eight of the participants had incomplete data, multiple imputation was used to estimate the missing values. The models using multiple imputation are summarized in Tables 5 and 6. As can be seen from tables, the odds ratio estimates for independent variables were fairly close whether or not multiple imputations were used. The lambda value in these tables represents the proportion of each estimate which is attributable to missing data.

**Table 5.** Dichotomized Katz model with multiple imputation.

	p	Nmis	Fmi	Lambda	Odds ratio
Age (years)	0.825	0	0.032	0.009	1.007 (0.947–1.070)
EMAS score	0.316	4	0.073	0.049	0.947 (0.850–1.054)
FCI score	0.162	1	0.051	0.028	0.860 (0.695–1.064)
GDS score	0.683	4	0.101	0.077	1.064 (0.788–1.437)

Katz: Katz Activities of Daily Living Index; EMAS: Engagement with Meaningful Activity Survey; FCI: Functional Comorbidity Index; GDS-SF: Geriatric Depression Scale–Short Form; Nmis: number missing; Fmi: fraction of missing information.

**Table 6.** Dichotomized Lawton–Brody model with multiple imputation.

	p	Nmis	Fmi	Lambda	Odds ratio
Age (years)	0.001	0	0.028	0.006	0.838 (0.769–0.914)
EMAS score	0.003	4	0.028	0.005	1.147 (1.011–1.302)
FCI score	0.287	1	0.024	0.001	0.871 (0.674–1.126)
GDS score	0.620	4	0.105	0.079	1.082 (0.789–1.484)

Lawton–Brody: Lawton–Brody Instrumental Activities of Daily Living Scale; EMAS: Engagement with Meaningful Activity Survey; FCI: Functional Comorbidity Index; GDS-SF: Geriatric Depression Scale–Short Form; Nmis: number missing; Fmi: fraction of missing information.

Goodness of fit for the models was tested by comparison of residuals with null models. Chi square for the dichotomized Katz model was χ^2^ (4, N = 86) = 5.549, p = 0.235. For the dichotomized Lawton–Brody, it was χ^2^ (4, N = 86) = 22.471, p < 0.001.

Two of the instruments are relatively new: the EMAS and the FCI. For the EMAS, Cronbach’s alpha for this study was 0.83, which is close to the 0.84 reported by the instrument developers.^57^ In contrast to Eakman et al.s’^55^ study that reported negative correlation between the EMAS and depression, there was no significant correlation between EMAS score and GDS-SF score in this study. FCI score was not significantly correlated with any study variable. The closest relationships to significance were with the GDS-SF (r = 0.158, p = 0.133) and with the Katz (r = –0.123, p = 0.243).

### Effect of engagement

The odds ratio for age in the Lawton–Brody model was 0.838. The inverse can be taken, which translates to an odds ratio of 1.193 for getting a less-than perfect Lawton–Brody score, meaning that for each additional year of age, the odds of getting an imperfect score increased by 19.3%. The odds ratio for the EMAS score is 1.147. For each one point increase in EMAS score, a participant’s odds of getting a perfect score on the Lawton–Brody increase by 14.7%.

### Lack of correlation for depression and comorbidity

One of the unexpected findings of this study was the lack of significant influence of the comorbidity and depression variables. FCI score was the variable which came closest to significance in the Katz model. This contrasts with the study by Marengoni et al.,^70^ which found that one comorbidity multiplied the odds by 1.4 and four or more comorbidities multiplied them by 6.2. The Marengoni study had a much larger sample than this study and was longitudinal.

GDS-SF score was not a significant predictor in either model. Correlations between GDS-SF and the Katz ADL and Lawton Brody IADL scores were r = 0.012 (p = 0.914) and r = –0.082 (p = 0.448), respectively. Mehta et al.^71^ found that depression was a risk factor for functional decline among functionally intact community-dwelling older adults but not for those who already had some functional decline. Since this study was cross-sectional, the more depressed participants could be at risk for future functional decline which is not yet apparent.

## Discussion

This study identified engagement as a factor associated with better IADL level of function, but it is unclear whether the participants were more engaged because they had better level of function or whether they had better level of function because they were more engaged. Future studies should be done using more objective and more sensitive tools to measure level of functional status. There is also a need for longitudinal studies concerning this relationship.

There were significant predictors in the Lawton–Brody model, but not in the Katz model. Both of these instruments had ceiling effects in this study. The Lawton–Brody measures more complex skills such as doing light housework or arranging for transportation. This suggests that some of the older adults in this study were in the earliest stages of functional decline which was captured by the Lawton–Brody scores but not by the Katz scores.

Glass et al.^72^ studied the relationship between social engagement and depression among community-dwelling older adult participants in a population-based study. They found that social engagement was protective against the development of depressive symptoms and hypothesized three possible mechanisms: that social engagement may stimulate body functions such as cardiovascular or endocrine functions, that social engagement may slow cognitive decline, and that social engagement may be a proxy for coping ability. These three mechanisms postulated by Glass et al. could also play a role in the relationship between engagement and IADL level of function.

Hall et al.^73^ developed a theory which postulates both engagement strategies and disengagement strategies in response to health challenges. Engagement strategies involve striving to maintain active control. Disengagement strategies involve giving up control in areas that one can no longer control in the interests of self-protection. Although engagement strategies are associated with greater longevity following acute events such as myocardial infarction or stroke, they are maladaptive when directed to the burden of chronic illness. Disengagement strategies are protective in the presence of chronic illness but maladaptive in the case of acute events. This could mean that when prized goals and activities are unattainable, it is more adaptive to revise expectations than to persist in futile striving. In relation to this study, engagement could be helpful in the case of a patient with mobility limitations who needs motivation to exercise regularly, but it would not be helpful in the case of a patient with no realistic possibility of recovery.

Kratz et al.^74^ studied chronic pain in patients with neurological disorders such as multiple sclerosis and found that activity engagement predicted better adjustment to living with chronic pain than did pain willingness. Pain willingness is the amount of pain a person will accept. Although both of these variables are associated with positive adjustment to life with chronic pain, activity engagement is a more robust predictor. These findings echo those of Hall et al. mentioned above. Pain acceptance represents a disengagement strategy and activity engagement represents an engagement strategy.

### Limitations

Limitations of this study include its reliance on convenience sampling and self-report data. Since the sample was skewed toward women and toward White/Caucasian participants, the findings may not be applicable to other groups. The participants were recruited from different sites, and there may have been site-dependent variation in their functional ability. The Lequerica and Kortte’s^4^ model included environment as one of its concepts, which is a further argument for having controlled the environment variable. The construct of interest was engagement, but the participation component of engagement was not measured by the tool which was used. There were pronounced ceiling effects for both measures of functional status. Some variables of interest such as cognitive status, socioeconomic status, educational level, and social support were not measured.

This study was limited to participants with sufficient cognitive function to verbalize understanding of the consent document. Although some participants may have had mild dementia, it was not noticeable. Further study is needed to determine whether the relationship between engagement and IADL level of function holds true for older adults with dementia. The study procedures would also have to be modified to accommodate participants with dementia.

### Implications for research and practice

Nurses working with older adults might consider facilitating engaged activity for their patients. One way of doing this is to consider a patient’s former accustomed roles and to offer a patient role-congruent activities. Activity which activates the accustomed roles of the patient is more likely to be meaningful and thus to elicit higher levels of engagement. For example, a former executive might balk when asked to do arts and crafts but might respond more favorably when offered the opportunity to manage a group activity. Malone and Camp^75^ give an example of how to engage a reluctant client in personally meaningful activity. A therapist knew that a client had formerly been a gardener, so she invited the client to arrange flowers with her. Initially, the client refused, so the therapist asked whether she could stay in the client’s room and arrange the flowers herself. The client agreed to this. The therapist continued to approach the client daily without pushing her unduly. Eventually, the client began to offer suggestions and to start doing the activity herself.

Health-care professionals should also assess clients’ levels of engagement with activity to better identify clients at increased risk for functional decline. This can be done using formal tools such as the EMAS or may be done qualitatively by observing for levels of commitment, motivation, and participation.
